# Supplementary material for: Central Serous Chorioretinopathy by Autofluorescence, Enface and SLO–Retromode Imaging
Source: Life (Basel). 2023 Jun 17;13(6):1407. doi: 10.3390/life13061407 (PMC10304114; doi:10.3390/life13061407)
Supplement: Supplementary file 1 [file life-13-01407-s001.zip › life-2367555-supplementary.pdf]

Statistical Analysis Plan

The sample was described in its clinical and demographic characteristics by descriptive statistics techniques. In depth, qualitative data were expressed as absolute and relative percentage frequency, whilst quantitative as either mean and standard deviation (SD) or median and interquartile range (IQR), as appropriate. To verify the Gaussian distribution of quantitative variables, the Shapiro-Wilk test will be applied. Missing values, <5% in all cases, were treated by *imputeR* R package, using multiple imputation with Lasso Regression methods centered on the mean as for quantitative data, whilst classification trees for imputation by "rpartC" function, centered on the mode, i.e. most represented class object, were applied on qualitative data (S1).
Comparison between the different areas were performed using a Wilcoxon rank-sum test for paired data, whilst the two diagnostic techniques outcomes were compared by the Mc Nemar test.
Spearman's correlation test was applied to assess the correlation between the variables of interest. Spearman's rho coefficient, 95% confidence interval (CI) and p-value were further reported. The analysis was performed with *Hmisc* and *reshape2* R packages (S2,S23). A heat-map, drawn with "*ggplot2*" R package, further depicted the observed correlations (S4).
Accuracy of retromode imaging as compared to autofluorescence was assessed by a ROC Curve analysis performed with "*pROC*" R package (S5). Area Under the ROC curve (AUROC) and 95% confidence interval, specificity, sensitivity, negative and positive predictive value (NPV and PPV) were further reported, alongside with the ROC Curve itself.
Statistical significance was set at a p-value <0.05. Suggestive p-values were further reported (0.05≤p<0.10). All analyses were conducted with R software v4.2.0 (CRAN ®, R Core 2022, Wien, Austria) (S6).

References

S1. Feng L, Moritz S, Nowak G, Welsh AH, O'Neill TJ (2020). _imputeR: A General Multivariate Imputation Framework_. R package version 2.2, https://
CRAN.R-project.org/package=imputeR.

S2. Harrell Jr F (2022). _Hmisc: Harrell Miscellaneous_. R package version 4.7-2, https://CRAN.R-project.org/package=Hmisc

S3. H. Wickham. Reshaping data with the reshape package. Journal of Statistical Software, 21(12), 2007.

S4. H. Wickham. ggplot2: Elegant Graphics for Data Analysis. Springer-Verlag New York, 2016.

S5. Robin X, Turck N, Hainard A, Tiberti N, Lisacek F, Sanchez JC and Müller M (2011). pROC: an open-source package for R and S+ to analyze and compare ROC curves. BMC Bioinformatics, 12, p. 77. doi: 10.1186/1471-2105-12-77

S6. R Core Team. R: A language and environment for statistical computing https://www.R-project.org/. R Foundation for Statistical Computing, Vienna, Austria. 2022.

Supplementary Table S1. Correlation analysis (N=32).

	Age	Visual acuity	AF area	RM area	Enface area
Age	-				
Visual acuity (LogMar)	**-0.63 (-0.83; -0.36); <0.001**	-			
AF area (mm²)	**0.46 (0.12;0.72); 0.007**	*-0.30 (-0.56; -0.00); 0.091*	-		
RM area (mm²)	**0.35 (-0.05; 0.63); 0.046**	-0.21 (-0.48; 0.10); 0.250	**0.91 (0.81; 0.96); <0.001**	-	
Enface area (mm²)	**0.45 (0.08; 0.73); 0.009**	**-0.43 (-0.68; -0.11); 0.014**	**0.82 (0.64; 0.91); <0.001**	**0.82 (0.61; 0.94); <0.001**	-

* Data are reported as rho (95% confidence interval and p-value). In bold significant findings and in italic the suggestive ones.
